# Knowledge, Attitudes, and Practices Regarding COVID-19 Among Healthcare Workers in Venezuela: An Online Cross-Sectional Survey

**DOI:** 10.3389/fpubh.2021.633723

**Published:** 2021-07-13

**Authors:** Daniela Lucía Mendoza Millán, Fhabián Stevens Carrión-Nessi, Mario Daniel Mejía Bernard, María Victoria Marcano-Rojas, Óscar Daniel Omaña Ávila, Juan Manuel Doval Fernández, Fabián Rafael Chacón Labrador, Adriana Quintero Rodríguez, Sebastian Gasparini Vega, Adriana Tami, Andrea L. Maricuto, Viledy L. Velásquez, María Eugenia Landaeta, Manuel Figuera, Melynar Chavero, Luisamy Figuera, Natasha A. Camejo-Ávila, David A. Forero-Peña

**Affiliations:** ^1^“Luis Razetti” School of Medicine, Central University of Venezuela, Caracas, Venezuela; ^2^Biomedical Research and Therapeutic Vaccines Institute, Ciudad Bolívar, Venezuela; ^3^University of Groningen, University Medical Center Groningen, Department of Medical Microbiology and Infection Prevention, Groningen, Netherlands; ^4^Faculty of Health Sciences, University of Carabobo, Valencia, Venezuela; ^5^Infectious Diseases Department, Caracas University Hospital, Caracas, Venezuela

**Keywords:** COVID-19, healthcare workers, knowledge, attitude, practice study, health system, Venezuela, pandemic

## Abstract

**Background:** The deterioration of Venezuela's health system in recent years undoubtedly contributes to an increased impact of the COVID-19 pandemic. Understanding healthcare workers' (HCWs) knowledge, attitudes, and practices (KAPs) toward COVID-19 in the early stages of the pandemic could inform their medical training and improve their preparedness.

**Methods:** A online national cross-sectional survey was conducted between May 26th and May 30th, 2020, to assess KAPs among HCWs in Venezuela.

**Results:** A total of 1,441 HCWs from all 24 regions of the country responded to the survey. The mean age of the HCWs was 44 (*SD* [standard deviation] 14) years; most were women (66.4%). Most HCWs were specialized doctors (48%), followed by nurses (13%) and resident doctors (12.3%). The majority of HCWs had good knowledge (76.3%), obtained information mainly from scientific literature (85.4%); had negative attitudes (53.6%), felt uncomfortable with their work during the current pandemic (59.8%); and reported appropriate practices (76.9%). However, participation in COVID-19 related training was absent in more than half of the HCWs. Positive attitudes were significantly more frequent in frontline workers than in non-frontline workers (*p* = 0.001). Bioanalysts, students, and doctors were more likely to have good knowledge; participating in training was a predictor for positive attitudes and older age was an appropriate practice predictor.

**Conclusions:** HCWs, knowledge in Venezuela could be improved by strengthening education and training programs. Strategies should focus on reducing fear and improving attitudes toward the care of COVID-19 patients, as well as the promotion of preventive practices.

## Introduction

Coronavirus disease 2019 (COVID-19) is an emerging public health problem that threatens millions of lives worldwide. It is caused by the severe acute respiratory syndrome coronavirus two (SARS-CoV-2), first detected in Wuhan City, Hubei Province, China at the end of 2019 (1). COVID-19 spread throughout all continents showing an exponential growth, and was declared as pandemic on March 11th, 2020 by the World Health Organization (WHO) (2). By April 21st, 2020, a total of 2,397,217 cases were reported worldwide (3), of which around 35,000 (1.5%) cases were affecting healthcare workers (HCWs). Up to now, the WHO does not systematically report COVID-19 cases in HCWs and therefore this estimate is probably underrepresented (4).

Prevalence of infection in the early stage of the disease outbreak in HCWs reaches 1% in Tongji Hospital, Wuhan, China (5); however, in two hospitals in southern the Netherlands was higher than 6% (6). In the United States (7) and Italy (8) are reported a prevalence of SARS-CoV-2 infection in HCWs that vary between 7 and 11%. COVID-19 represents an occupational health risk among HCWs due to their frequent exposure to infected individuals. HCWs of all levels and groups have a higher SARS-CoV-2 infection rate than the rest of the population (9–12); however, studies on infection rate factors among HCWs are scarce and have methodological limitations that include poor control of intensity and frequency of exposure and of confounding factors (13).

Transmission between HCWs is associated with overcrowding, lack of isolation room facilities, and environmental contamination (14), likely exacerbated because some HCWs have inadequate knowledge and practices of infection prevention (15). Several studies have reported adequate knowledge among HCWs regarding COVID-19 (16–18); however, others have reported gaps in knowledge and inappropriate practices (19–21). HCWs' lack of solid knowledge related to COVID-19 can lead them to inappropriate practices, to overestimate the situation, increase their stress and anxiety, and disrupt the adequacy of their medical judgments. A knowledge, attitudes, and practices (KAPs) survey is a suitable way to evaluate existing programs and to identify effective strategies for behavioral change.

South America's health systems are particularly vulnerable given their poor response capacity (21). Venezuela is one of the most vulnerable in the region due to its current economic and health crisis (22, 23). Determining HCWs' KAPs in the early stages of the pandemic could inform HCWs' baseline training in Venezuela, to subsequently design strategies aimed at decreasing the unknowns and misfeasance of HCWs. To the best of our knowledge, there are no studies evaluating KAPs on HCWs regarding COVID-19 in Venezuela. We performed an online national cross-sectional survey in HCWs of all levels during the early phase of the COVID-19 outbreak in Venezuela.

## Methods

### Study Design, Participants, and Sample Size Calculation

We conducted an online national cross-sectional survey between May 26th and May 30th, 2020, using the “Google Forms”® platform. Due to the COVID-19 pandemic restrictions, we distributed the link to the online survey through the “WhatsApp”® application to the main verified official groups of the different institutions/unions/societies related to health. Likewise, the survey was distributed through social networks of the Venezuelan Association of Infectious Diseases. The survey was voluntary, anonymous, and confidential. Data about the current national number of HCWs in the country is not available, but given the online national cross-sectional nature of the study a population size of 20,000 was used to calculate the sample size with confidence interval of 95%, and margin of error of 5%, a total number of 384 HCWs was obtained. The sampling method was non-probabilistic.

### Ethical Approval

All procedures performed in this study, involving human participants, complied with the ethical standards of the relevant national and institutional committees on human experimentation and with the *Helsinki Declaration* of 1975, as revised in 2008. The study protocol was reviewed and approved by the National Center for Bioethics (CENABI, in Spanish) of Venezuela (CIBI-CENABI-14/2020). After explaining the nature of the study and the characteristics of the survey, only people who voluntarily agreed to participate in the study and gave their informed consent online, were included.

### KAPs and Socio-Demographic Evaluation

We designed a survey instrument based on guidelines, reports, and course material regarding emerging respiratory diseases, including COVID-19 by the WHO (24–28). The language of the questionnaire was in the official language of Venezuela (Spanish). The questionnaire was validated by professionals from medical backgrounds (infectious diseases specialists and epidemiologists) who gave their expert opinion with respect to its simplicity and importance. The questionnaire consisted of 30 questions assessing demographics, KAPs toward COVID-19, and whether they worked as a frontline COVID-19 health provider (Appendix in the Supplementary Material). Demographic characteristics included were age, sex, education, and institution. The knowledge section had 12 items about symptoms, treatment, and prevention of COVID-19; correct answers scored 1 and incorrect answers scored 0, total score range from 0 to 12 points. The attitudes section had five items about confident or fearful behavior toward COVID-19, and responses were recorded on a five-point Likert scale (1, strongly agree; 2, agree; 3, undecided; 4, disagree; 5, strongly disagree), with a score range from 5 to 25 points. The final section about prevention practices regarding COVID-19 had four items, each item was recorded on a five-point Likert scale (1, never; 2, rarely; 3, sometimes; 4, frequently; 5, always), with a score range from 4 to 20 points. Better knowledge, attitudes, or practices are indicated by higher points in each section. Responses are presented as frequencies and percentages, given that the scales measure completely different dimensions, it was considered that a general result (total) was not appropriate, and thus the individual analysis of each dimension was chosen and presented.

### Survey Validation

This questionnaire was pilot tested in 72 HCWs, with a mean age of 41 (*SD* [standard deviation] 12) years and predominantly women (*n* = 47; 65.3%), one month before its application. On the “knowledge” dimension, the results were dichotomous (correct: 1/incorrect: 0) and analyzed by two-parameters Item Response Theory. Difficulty range between −3 to +3 and discrimination range higher than 0.25, were considered acceptable. The “attitudes” and “practices” dimensions were submitted to exploratory factor analysis (principal components); we used Kaiser-Meyer-Olkin test and Bartlett's test of sphericity to demonstrate the factorization, taking into consideration a factor loading ≥0.45 as significant.

### Statistical Analysis

The data analysis considered descriptions of the characteristics through the use of central tendency and dispersion measures (mean and standard deviation). We evaluated parameter distribution using Kolmogorov–Smirnov test. A latent class analysis was used to exhibit the existence of non-evident (latent) groups within the sample in question. Applying the Bayesian Information Criterion, it was determined that two classes were optimal for each dimension of the questionnaire. Each individual was then paired in one of the two classes according to their means and marginal probabilities. A Receiver Operating Characteristic curve identified cutoff points for each dimension, taking into account the percentage of correctly classified individuals. Finally, once the cutoff points were obtained, HCWs were classified in one of two categories; for the knowledge dimension: poor/good, for the attitudes dimension: positive/negative, and for the practices dimension: appropriate/inappropriate. Through binary logistic regression, we determined possible predictor factors for better or worse scores on KAPs. The following variables were included in the model: age, gender, profession, frontline or non-frontline worker, and source of acquired knowledge. As well, categorical variables were analyzed using Chi-square and Fisher's exact tests, and quantitative variables were analyzed using Student's *t*-test. The data was processed in IBM SPSS® v.25.0 and STATA v.16. Figures were performed using Microsoft® Excel® version 2013, and map was performed using Microsoft® Excel® version 2019 (Microsoft, Redmond, WA, United States). A *p* < 0.05 was considered significant.

## Results

**Figure 1:**
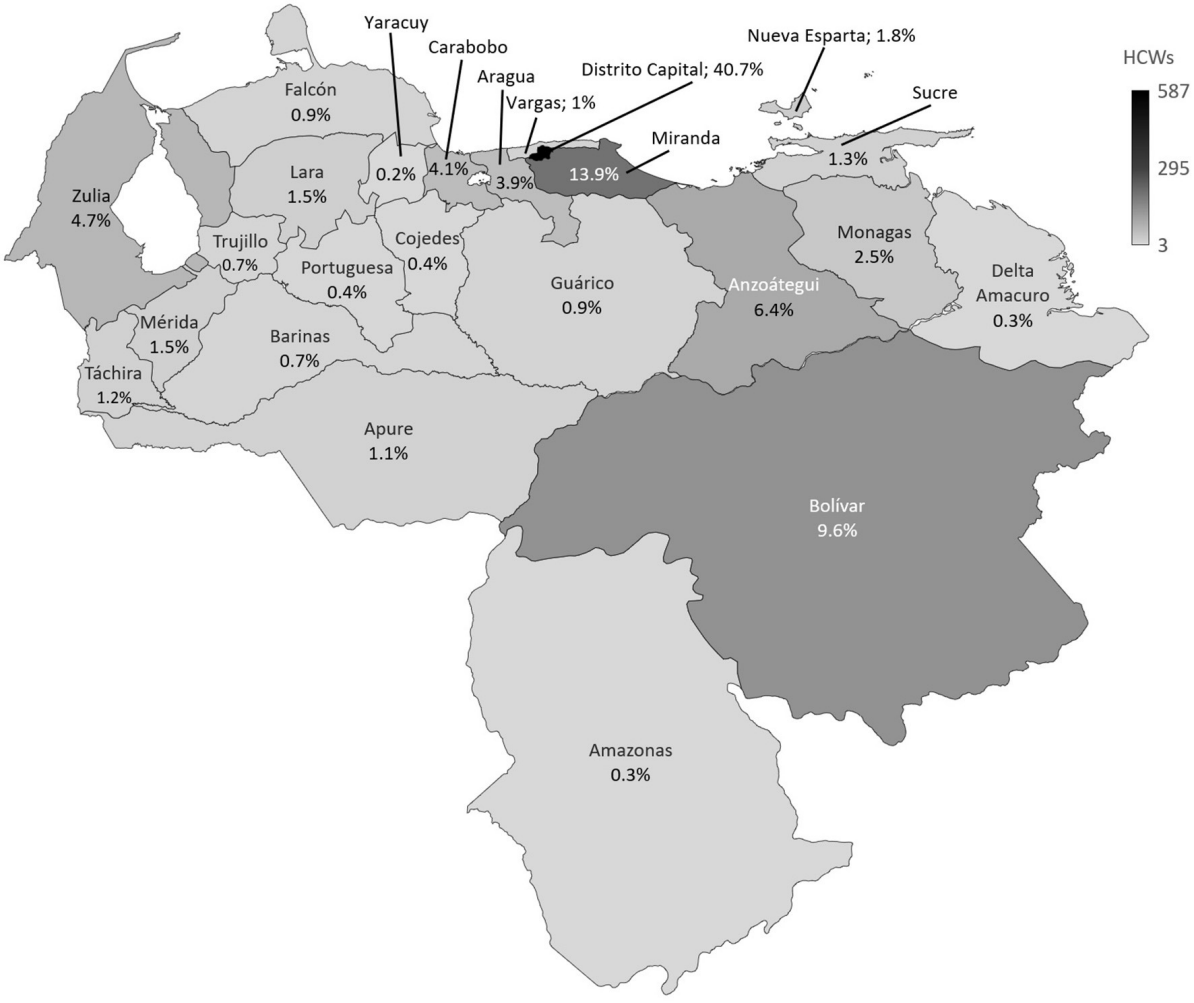
Healthcare workers from the 24 states of Venezuela that responded to the survey. Number of healthcare workers surveyed is represented in gray scale. Frequency of healthcare workers surveyed is represented in percentage within each state. Map was performed using Microsoft® Excel® version 2019 (Microsoft, Redmond, WA, United States).

A total of 1,441 HCWs living in the 24 states of the country responded to the survey (Figure 1). The mean age was 44 (*SD* 14; range 20–90 years) years old, most of the HCWs were women (*n* = 957; 66.4%). Most HCWs were specialized doctors (*n* = 694; 48.2%), followed by nurses (*n* = 187; 13%) and resident doctors (*n* = 177; 12.3%). Most of the HCWs responding to the survey (*n* = 990; 68.7%) worked in public health centers, just 30.2% (*n* = 435) worked in private ones. The main source of knowledge about COVID-19 was through the scientific literature (*n* = 1,230; 85.4%), colleagues (*n* = 789; 54.8%), and social networks (*n* = 697; 48.4%) (Table 1). Only 471 of those surveyed (32.7%) had areas for the care and management of patients with COVID-19 in their working institution (716 [49.7%] did not have, 254 [17.6%] did not answer).

**Table 1:** Healthcare workers' socio-demographic characteristics and source of knowledge (*n* = 1,441).

**Demographic variables**	***n***	**%**
**Age groups (years)**		
18–27	211	14.6
28–37	364	25.3
38–47	216	15
48–57	364	25.3
≥58	286	19.8
**Gender**		
Female	957	66.4
Male	484	33.6
**Profession in the health field**		
Specialized doctor	694	48.2
Nurse	187	13
Resident doctor	177	12.3
General doctor	126	8.7
Dentist	68	4.7
Licentiate (master) in bioanalysis	66	4.6
Student	43	3
Technician	25	1.7
Pharmacist	9	0.6
Nutritionist	9	0.6
Integral community doctor	8	0.6
Psychologist	7	0.5
Others^*^	22	1.5
**Frontline worker for COVID-19**		
No	975	67.7
Yes^†^	466	32.3
**Sources of knowledge**		
Scientific literature	1,230	85.4
Colleagues	789	54.8
Instagram/Twitter/WhatsApp/Facebook	697	48.4
TV/Radio/Newspaper	459	31.9
Training at the health center where I work	339	23.5
Webinar/Online courses	84	5.8
Friends/Neighbors	76	5.3
Internet/Google/Online news/YouTube	35	2.4

* *Hemotherapist, medical records, medical histories, public health, occupational therapy, biologist, microbiologist, social work, physiotherapist, paramedic*.

† *Isolation in a unique room or with at least six feet distance between each bed, correct deposition of biosecurity waste, etc*.

### HCWs' Knowledge Related to COVID-19

Good knowledge was defined as ≥7 points [*area under the curve* (AUC) = 1; sensitivity: 100%; specificity: 100%]. Good knowledge was observed in the majority of the HCWs (*n* = 1,100; 76.3%); however, there was poor knowledge regarding the virus name (*n* = 906; 62.9% chose the wrong answer), and proper use of personal protective equipment (PPE) in different scenarios (mistakes were noted in 1,431 HCWs [99.3%] for triage, 835 [57.9%] for aerosol-generating procedures, and 1,327 [92.1%] for attending a patient without aerosol-generating procedures) (Table 2).

**Table 2:** Healthcare workers' knowledge related to COVID-19.

**Knowledge**	**Answer** ***n*** **=** **1,441 (100%)**		
	**Correct**	**Incorrect**	**Unknown**
**General**			
The virus name is COVID-19.	531 (36.8)	906 (62.9)	4 (0.3)
The incubation period of COVID-19 is from 2 to 14 days.	1,345 (93.3)	90 (6.2)	6 (0.4)
Fever, cough, and shortness of breath are common symptoms of COVID-19.	1,404 (97.4)	34 (2.4)	3 (0.2)
Antibiotics are the first line of treatment for COVID-19.	1,278 (88.7)	95 (6.6)	68 (4.7)
Oseltamivir is an effective treatment for COVID-19.	730 (50.7)	203 (14.1)	508 (35.3)
Gargling with warm water is recommended as prophylaxis for COVID-19.	1,065 (73.9)	273 (18.9)	103 (7.1)
Quarantine means to restrict for 14 days the movement and contacts of a healthy person that has been exposed to an infected person with COVID-19.	994 (69)	440 (30.5)	7 (0.5)
Isolation means keeping a sick person separated from healthy persons during the infectious period.	1,367 (94.9)	70 (4.9)	4 (0.3)
Health-care workers have higher risk of infection from COVID-19.	1,377 (95.6)	57 (4)	7 (0.5)
**Type of protection in different scenarios**			
Triage of patients with respiratory symptoms.	10 (0.7)	1,431 (99.3)	–
Procedure that generates aerosols in a hospitalized patient due to COVID-19 (example: intubate).	606 (42.1)	835 (57.9)	–
Care and management of a hospitalized patient due to COVID-19 (excluding procedures that generate aerosols).	114 (7.9)	1,327 (92.1)	–

### COVID-19 Related Attitudes of HCWs

**Figure 2:**
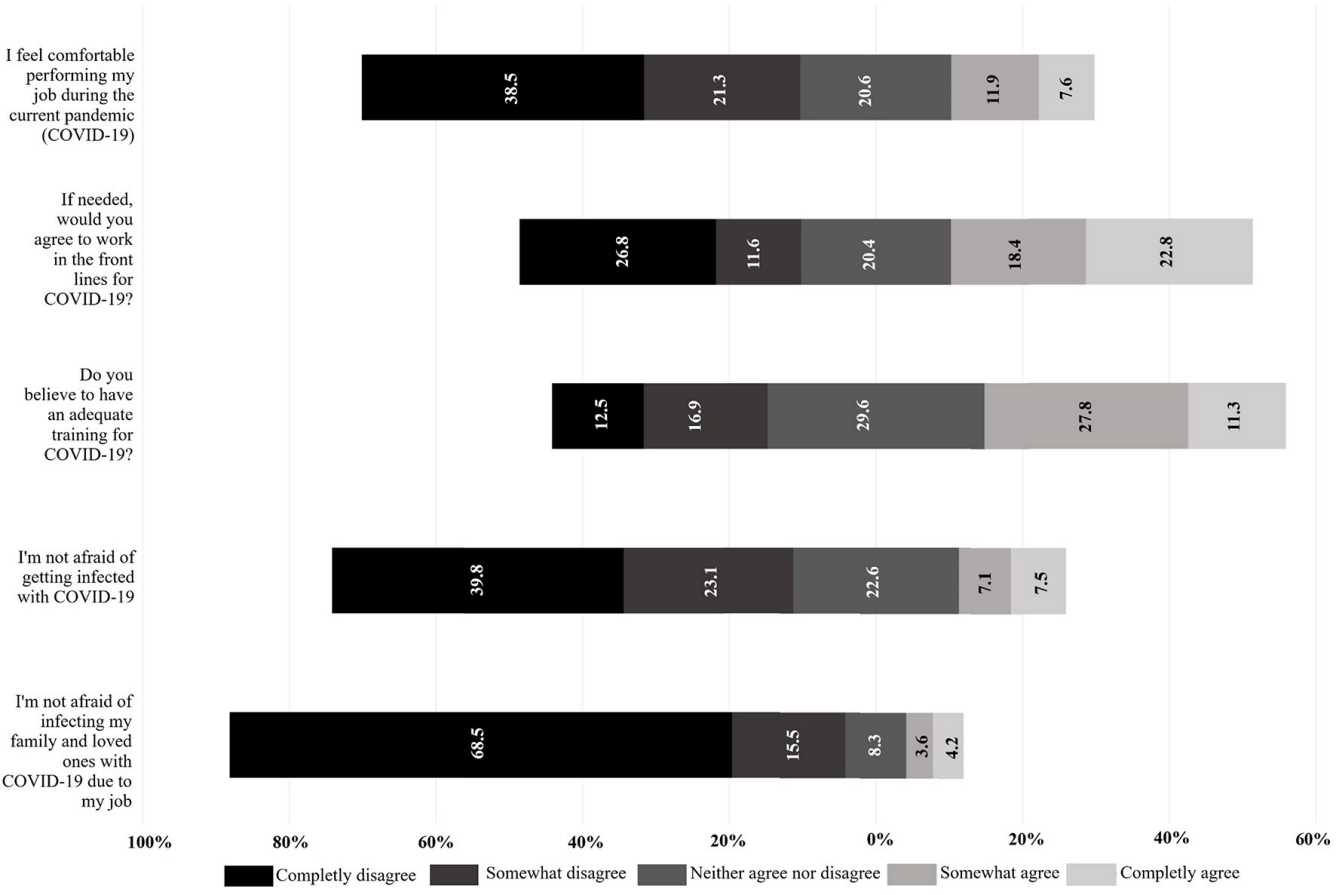
Attitudes regarding COVID-19 in healthcare workers. Piled Bars showing proportion of patients according to their choice for each item in the attitudes dimension. Better attitudes tend to the right; worse attitudes tend to the left. Indifferent attitudes (gray bars) are placed in the middle. Figure was performed using Microsoft® Excel® version 2013 (Microsoft, Redmond, WA, United States).

Positive attitudes were defined as ≥13 points [*AUC* = 0.84 (IC 95%: 0.81–0.86); sensitivity: 90.04%; specificity: 61.98%]. Negative attitudes were observed in the majority of the HCWs (*n* = 773; 53.6%) regarding COVID-19. Most of the HCWs were not comfortable with their work during the current pandemic (*n* = 862; 59.8%), only 39.1% (*n* = 563) of HCWs considered themselves to have adequate medical training related to COVID-19. Fear of becoming infected was frequent (*n* = 906; 62.9%), and fear of infecting their relatives and loved ones was even higher (*n* = 1,210; 84%) (Figure 2).

### Practices Related to COVID-19 in HCWs

**Figure 3:**
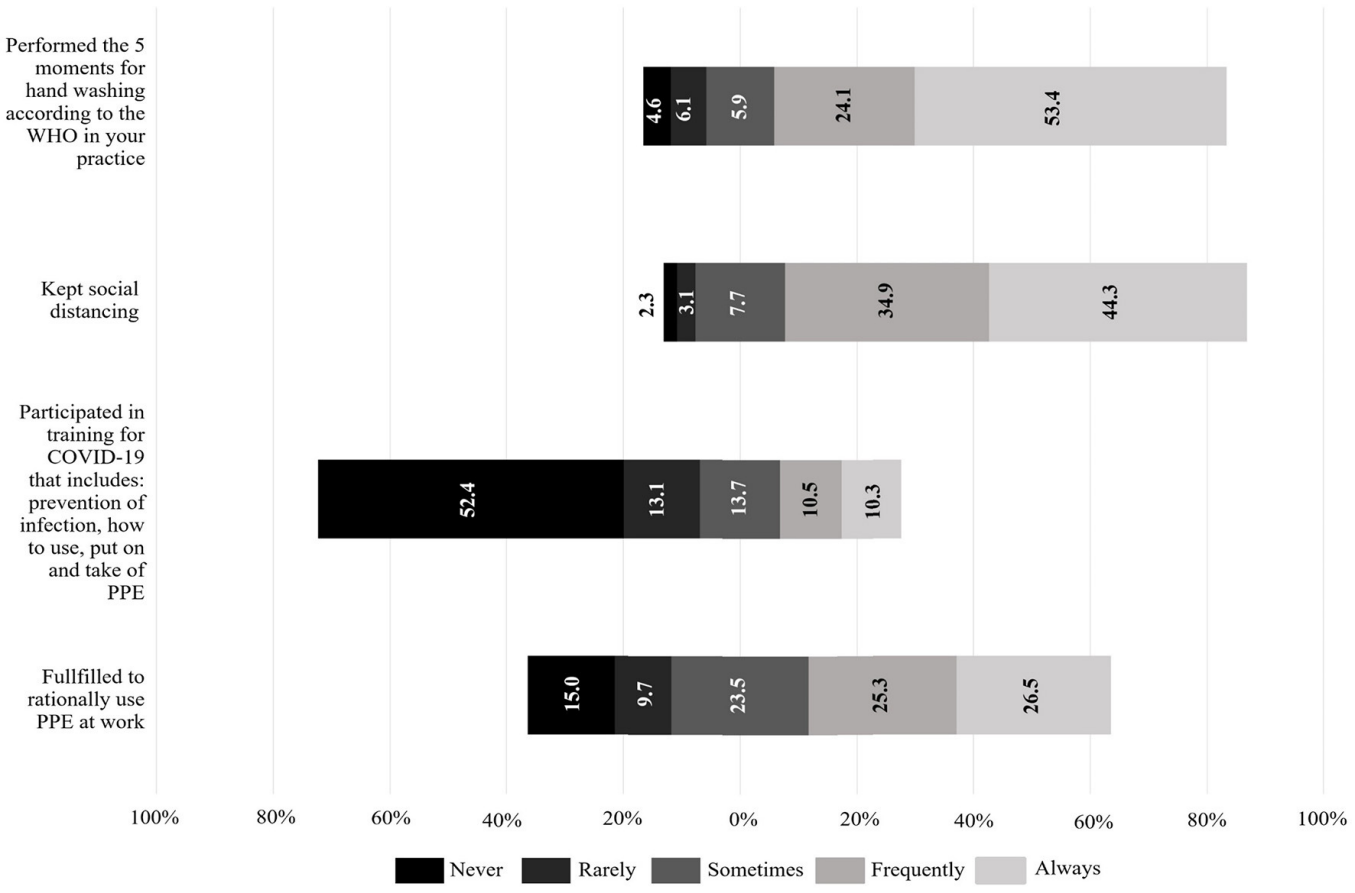
Practices related to COVID-19 in healthcare workers. Bars indicate the proportion of patients according to their choice for each item in the practices dimension. Better practices tend to the right, worse attitudes tend to the left. Gray area (neither agrees nor disagrees) is placed in the middle. Figure was performed using Microsoft® Excel® version 2013 (Microsoft, Redmond, WA, United States).

Appropriate practices were defined as ≥13 points (*AUC* = 1; sensitivity: 100%; specificity: 100%). Appropriate practices were observed in the majority of the HCWs (*n* = 1,108; 76.9%). Most of the HCWs always or frequently performed social distancing (*n* = 1,142; 79.2%), hand hygiene (*n* = 1,117; 77.5%), and rational use of PPE (*n* = 746; 51.8%); however, more than half of the HCWs report to have never participated in trainings about COVID-19 (*n* = 755; 52.4%), which include rational use of PPE (Figure 3).

### Frontline HCWs

Four hundred and sixty-six (32.3%) professionals indicated that they are frontline workers in COVID-19 areas; frontline workers were younger than non-frontline workers (mean age 40.4 vs. 45.7 years; *p* < 0.001). The nurses, general doctors, and resident doctors worked more frequently as frontline worker for COVID-19 (*p* < 0.001), while the specialized doctors and dentists were more frequent off the frontline. It was more frequent that HCWs working in the public health sector belonged to the frontline worker (*p* < 0.001). Good knowledge and appropriate practices prevailed in the HCWs, with no significant differences between frontline and non-frontline groups (Table 3). Positive attitudes were significantly more frequent in frontline workers (52.8%), whereas in non-frontline workers, negative attitudes were more frequent (56.7%) (*p* = 0.001). Most frontline workers (53.9%) agreed to work in the frontline against COVID-19, while less than half (35.2%) of non-frontline workers would agree, if they were asked to.

**Table 3:** Factors associated with knowledge, attitudes, and practices outcome.

**Characteristics**	**Knowledge**			**Attitudes**			**Practices**		
	**Poor (*n* = 341)**	**Good (*n* = 1,100)**	***p***	**Negative (*n* = 773)**	**Positive (*n* = 668)**	***p***	**Inappropriate (*n* = 332)**	**Appropriate (*n* = 1,109)**	***p***
**Gender**			0.01			<0.001			0.644
Male	95 (19.6)	389 (80.4)		228 (47.1)	256 (52.9)		115 (23.8)	369 (76.2)	
Female	246 (25.7)	711 (74.3)		545 (56.9)	412 (43.1)		217 (22.7)	740 (77.3)	
**Profession in the health field**									
Bioanalyst	10 (15.2)	56 (84.8)	0.096	24 (36.4)	42 (63.6)	0.004	10 (15.2)	56 (84.8)	0.119
Student	7 (16.3)	36 (83.7)	0.247	33 (76.7)	10 (23.3)	0.002	10 (23.3)	33 (76.7)	0.973
Nurse	86 (46)	101 (54)	<0.001	91 (48.7)	96 (51.3)	0.143	57 (30.5)	130 (69.5)	0.01
Specialized doctor	119 (17.1)	575 (82.9)	<0.001	381 (54.9)	313 (45.1)	0.357	123 (17.7)	571 (82.3)	<0.001
General doctor	27 (21.4)	99 (78.6)	0.537	72 (57.1)	54 (42.9)	0.41	38 (30.2)	88 (69.8)	0.059
Resident doctor	29 (16.4)	148 (83.6)	0.015	99 (55.9)	78 (44.1)	0.514	73 (41.2)	104 (58.8)	<0.001
Dentist	22 (32.4)	46 (67.6)	0.084	34 (50)	34 (50)	0.537	3 (4.4)	65 (95.6)	<0.001
Other	28 (50.9)	27 (49.1)	<0.001	11 (44)	14 (56)	0.329	14 (25.5)	41 (74.5)	0.744
Technician	13 (52)	12 (48)	0.001	28 (50.9)	27 (49.1)	0.678	4 (16)	21 (84)	0.481
**Frontline workers**			0.664			0.001			0.64
No	234 (24)	741 (76)		553 (56.7)	422 (43.3)		221 (22.7)	754 (77.3)	
Yes	107 (23)	359 (77)		220 (47.2)	246 (52.8)		111 (23.8)	355 (76.2)	

*The data is expressed in numbers and proportions, except the age where they are expressed in mean and standard deviation. The p-values are obtained by Chi2-square test except for age which were obtained by Student's t-test. Others: hemotherapist, medical records, public health, occupational therapy, biologist, microbiologist, social work, physiotherapist, paramedic*.

### Factors Associated With KAPs' Outcome

In general, most HCWs showed good knowledge, negative attitudes, and appropriate practices. Males tended to have better knowledge and attitudes than females (*p* = 0.01; *p* < 0.001; respectively). Good knowledge was common in most bioanalysts, students, and doctors (>80%). Negative attitudes prevailed in the surveyed population (*n* = 773; 53.6%), mostly in students (76.7% of them), and inappropriate practices were most frequent in resident doctors (41.2% of them).

Predictors for better score at knowledge (*R*^2^ *Nagelkerke* = 0.18; *p* < 0.001) were professions such as: bioanalyst (β: 1.8; 95% CI: 0.8–2.9; *p* = 0.001), student (β: 1.6; 95% CI: 0.5–2.8; *p* = 0.005), specialized doctor (β: 1.7; 95% CI: 0.9–2.5; *p* < 0.001), general doctor (β: 1.3; 95% CI: 0.4–2.2; *p* = 0.006), and resident doctor (β: 1.6; 95% CI: 0.7–2.5; *p* < 0.001), whereas for attitudes (*R*^2^ *Nagelkerke* = 0.063; *p* < 0.001), male sex (β: 0.5; 95% CI: 0.3–0.8; *p* < 0.001), working on the frontline (β: 0.4; 95% CI: 0.2–0.7; *p* = 0.001), participating in trainings (β: 0.3; 95% CI: 0.0–0.5; *p* = 0.043), and acquiring knowledge from scientific literature (β: 0.5; 95% CI: 0.1–0.8; *p* = 0.005) were predictors for positive attitudes, and a strong predictor for negative attitudes was being a student (β: 1.6; 95% CI: 0.5–2.7; *p* = 0.005). For practices (*R*^2^ *Nagelkerke* = 0.088; *p* < 0.001), older age was an appropriate predictor for good ones (β: 0.01; 95% CI: 0.00–0.03; *p* = 0.038); while being a resident doctor predicted lower scores (β: 1.2; 95% CI: 0.03–2.3; *p* = 0.044).

## Discussion

To our knowledge, this is the first national study that describes the KAPs of Venezuelan HCWs in relation to COVID-19. Most HCWs showed an overall good knowledge of COVID-19 (76.3%), as reported in other countries (17, 20, 29–33); however, important knowledge gaps were identified. Most of the HCWs could not identify the correct PPE for different scenarios with suspected or confirmed patients with COVID-19, frequently choosing overuse of PPE for low-risk scenarios, despite protocols widely described by organizations like the WHO (28). These decisions could be motivated by the high level of fear of infection that we report in this study.

Knowledge in our study was frequently based on scientific literature, colleagues, and social networks, similar to what is found in other studies (20, 31, 33). Nowadays, we can notice the positive impact in scientific knowledge from social networks, becoming everyday more popular and useful for scientific purposes; however, a professional approach is required due to the excess of unverified information through this network, especially during this rushed pandemic times (16, 17). We found knowledge acquired from scientific traditional literature predicted better attitudes toward COVID-19; therefore, granting access to it in a free, timely, and updated manner is urgently needed, where social networks could seem to fit.

Regarding the attitudes, most of the HCWs feared becoming infected with SARS-CoV-2 (62.9%), and feared infecting their relatives and loved ones even higher (84%). Similar results regarding fear of contagion have been observed in various studies; for example, in a study in Henan, China, where around 85% of the surveyed HCWs were afraid of becoming infected at work (18), and in Ho Chi Minh City, Vietnam, 82.3% of the surveyed HCWs were afraid of getting the disease, and 79.8% were afraid of infecting their loved ones (34). Compared to these studies, we found that Venezuelan HCWs are less afraid of becoming infected themselves, but their fear of infecting others was similar to that reported in the aforementioned studies. Furthermore, we observed that the majority of the surveyed personnel were not comfortable to perform their tasks during the current pandemic (59.8%). Venezuela suffers from a complex humanitarian crisis, their health system presents insufficient infrastructure, personnel and supplies, mentioned as “collapsed” previous to COVID-19 (35); in light of this recent pandemic, HCWs may present higher fear and restraint to work under these conditions, which imply a greater risk of infection.

According to their practices, similar to what was found in other studies (15, 17), only half of the HCWs always or frequently complied with rational use of the PPE. This may be correlated with the low knowledge about the WHO regulations for the rational use of PPE (28) documented in our knowledge assessment; additionally, this could relate with the scarce supply of PPE in health institutions, which is even limited in developed countries due to high global demand (36, 37). A study conducted in Latin America's HCWs, surveyed 20 countries in the region including Venezuela, reported limited access to the equipment recommended by the WHO, particularly disposable masks, N95 masks, and facial protective shields (38). In concordance, a meta-analysis from the United States showed HCWs not only lacked equipment, but they were concerned about feeling exposed to the virus due to the quality of it. Furthermore, in our study only half of HCWs always complied with the five times of hand hygiene recommended by the WHO in their daily practice, in contrast to another study that documents a higher percentage (76%) of compliance with this measure (17). This could be explained by the lack of access to water in most hospitals of the country, being the main reason why health providers cannot wash their hands (39, 40).

Infection prevention training for COVID-19 has been reported to be significantly associated with better knowledge scores and better practices (41), the absence or deficiency of training is a barrier to infection prevention and control practices adherence (42). Worryingly, we found that around half of HCWs had never participated in training programs on the prevention of COVID-19 and use, withdrawal and disposal of PPE. This suggests that there are limited training and education programs despite having passed more than two months since the first case in the country being; this is a worrying fact since we also found that training on COVID-19 predicts better attitudes. Similarly, most HCWs do not receive any training regarding mental health, especially in isolation units, leaving aside the possible anxiety disorders that confinement can cause in these workers, as it affects the rest of the population and increases their fear of the disease (43). With all these negative attitudes in consideration, only 41.2% (22.8% completely agree plus 18.4% somewhat agree) of the surveyed would work in the frontline if the situation in Venezuela requires it.

When comparing frontline workers against non-frontline workers for COVID-19, we found mean age was lower in the frontline workers, probably because older age has been identified as a risk factor for worse COVID-19 outcomes (44) and therefore older personnel should avoid exposure. We could also associate this with why specialized doctors, who tend to be older, remain off the frontline. Resident doctors and general doctors work more frequently on the frontline, consistently with results obtained in Jordan (45), in our case primarily because most hospitals with educational programs and primary care centers in Venezuela have been selected as sentinel centers for COVID-19. Older age was a strong predictor for better practices and being a resident doctor for inappropriate practices, probably associated with the precarious conditions in which frontline workers (who tend to be residents) have worked in this current situation. We found polemic attitudes in the HCWs, while most frontline workers tend to have positive attitudes regarding COVID-19 and therefore predict better scores for this section, most non-frontline workers presented negative ones, this could be explained as more frontline workers consider having adequate training related to COVID-19 (positive predictor for attitudes) and face the real danger of this disease day to day. Yet, only 53.9% of frontline workers agreed to work in their current position, feeling uncomfortable could affect their performance and increase their chances of getting infected (39).

### Study Strengths and Limitations

We used the WHO guidelines, reports, and training material regarding emerging respiratory diseases, including COVID-19, to develop a questionnaire. This questionnaire was pilot tested and subsequently validated by infectologists and epidemiologists to limit open-ended questions and reduce information bias. However, this study had some limitations that should be considered. This cross-sectional study was conducted online and the responses were not supervised, thus the data presented in this study is self-reported and partly dependent on the HCWs' honesty, suffered from social desirability bias and voluntary enrollment. The sampling method was non-probabilistic. Also, HCWs needed access to the internet to participate in the study and in many states and rural areas of the country there is little to no internet service, this could explain the fact that most of the HCWs were from the Capital District. Lastly, an aspect not evaluated during the cross-sectional study was the experience of the HCWs had with previous pandemics, which could determine better knowledge, attitudes and practices toward handling these situations. Despite these limitations, our findings provide valuable information about the knowledge, attitude, and practices of HCWs in Venezuela previous to the peak of the pandemic.

## Conclusion

This is the first KAPs study that shows the misconceptions of Venezuelan HCWs, as well as their attitudes toward the pandemic, which in turn influence practices. HCWs' knowledge in Venezuela could be improved, by strengthening education and training programs. Strategies should be focused on reducing fear and improving attitudes toward the care of COVID-19 patients, as well as the promotion of preventive practices.

## Data Availability Statement

The raw data supporting the conclusions of this article will be made available by the authors, without undue reservation.

## Ethics Statement

The studies involving human participants were reviewed and approved by National Center for Bioethics (CENABI, in Spanish), Faculty of Medicine, Central University of Venezuela, Caracas, Venezuela. The patients/participants provided their written informed consent to participate in this study.

## Author Contributions

DM and DF-P conceived and designed the study. DM, MM, and DF-P wrote the initial questionnaire. AT, ML, and MF edited and validated the same. ÓO collected and organized data. MM-R analyzed the data. DM, MM, FC-N, MM-R, JD, AQ, MC, LF, AM, and VV interpreted and discussed the data. FC-N wrote an initial draft of the manuscript. FC-N, FC, NC-A, and SG provided logistic support. All authors have critically reviewed and approved the final draft, and are responsible for the content and similarity index of the manuscript.

## Conflict of Interest

The authors declare that the research was conducted in the absence of any commercial or financial relationships that could be construed as a potential conflict of interest.
