# Supplementary material for: Knowledge, Attitudes, and Practices Regarding COVID-19 Among Healthcare Workers in Venezuela: An Online Cross-Sectional Survey
Source: Front Public Health. 2021 Jul 13;9:633723. doi: 10.3389/fpubh.2021.633723 (PMC8315295; doi:10.3389/fpubh.2021.633723)
Supplement: Supplementary file 1 [file Table_1.DOCX]

Supplementary Material

# QUESTIONNAIRE: Knowledge, attitudes, and practices among healthcare workers regarding COVID-19.

**Introduction:** The purpose of the following survey is to evaluate Venezuelan health-care workers’ preparation for the COVID-19 pandemic; it consists of 4 sections: general data, knowledge, attitudes, and practices regarding COVID-19. This is the instrument used for collecting data for the researched titled “Knowledge, attitudes and practices related to COVID-19 of the health-care workers in Venezuela”, executed by researchers from “Luis Razetti” Medical School, Central University of Venezuela, and from the Department of Infectious Diseases of the Adult, University Hospital of Caracas. This survey must only be answered by health-care workers, by completing it, you will be agreeing to use the collected information for scientific purposes, which will be completely anonymous and confidential.

- Are you a health care worker? **Yes/**No answer*

*“No” answer ends the survey.

Knowledge

Regarding the following statements:

1. The virus’ name is COVID-19: True/**False**/Unknown answer
2. The incubation period of COVID-19 is from 2 to 14 days: **True**/False/Unknown answer
3. Fever, cough and shortness of breath are common symptoms of COVID-19: **True**/False/Unknown answer
4. Antibiotics are the first line of treatment for COVID-19: True/**False**/Unknown answer
5. Oseltamivir is an effective treatment for COVID-19: True/**False**/Unknown answer
6. Gargling with warm water is recommended as prophylaxis: True/**False**/Unknown answer
7. Quarantine means to restrict for 14 days the movement and contacts of a healthy person that has been exposed to an infected person with COVID-19: **True**/False/Unknown answer
8. Isolation means keeping a sick person separated from healthy persons during the infectious period: **True**/False/Unknown answer
9. Health-care workers have a higher risk of COVID-19 infection: **True**/False/Unknown answer
10. Indicate which Personal Protection Equipment (PPE) do you consider necessary for the following scenarios; you may check one or more options following the guidelines set by the World Health Organization

| Scenario | Surgical face mask | N95, FPP2 or similar respirator | Surgical coat or suit | Eye protection (goggles or shield) | Gloves | None of the previous |
| --- | --- | --- | --- | --- | --- | --- |
| Triage of patients with respiratory symptoms (fever, cough, shortness of breath) | (Correct answer) |  |  | (Correct answer) |  |  |
| Procedure that generates aerosols in a hospitalized patient due to COVID-19 (example: intubate) |  | (Correct answer) | (Correct answer) | (Correct answer) | (Correct answer) |  |
| Care and management of a hospitalized patient due to COVID-19 (excluding procedures that generate aerosols) | (Correct answer) |  | (Correct answer) | (Correct answer) | (Correct answer) |  |

Attitudes

According to the following scale: 1= completely disagree; 2= somewhat disagree; 3= neither agree nor disagree; 4= somewhat agree; 5= completely agree; in your opinion:

1. I feel comfortable performing my job during the current pandemic (COVID-19): 1-5 answer.
2. If needed, will you agree to work at the frontlines for COVID-19 (having direct contact with suspicious or confirmed cases at the triage, consultations, taking and/or analyzing samples, transport, treatment, care or hospitalization)?: 1-5 answer.
3. Do you believe that you have adequate training regarding COVID-19?: 1-5 answer.
4. I’m not afraid of getting infected with SARS-CoV-2: 1-5 answer.
5. I’m not afraid of infecting family and loved ones with SARS-Cov-2 virus due to my job: 1-5 answer.

Practices

According to the following scale: 1= never; 2= rarely; 3= sometimes; 4= frequently; 5= always, you’ve:

1. Rationally used PPE at work: 1-5 answer.
2. Participated in training for COVID-19 that includes: prevention of infection, how to use, put on, and remove the PPE: 1-5 answer.
3. Kept social distancing: 1-5 answer.
4. Performed the 5 moments for hand washing according to the WHO in your practice (before contacting a patient, before an aseptic procedure, after being exposed to body fluids, after contact with a patient, after contacting with a patient’s environment): 1-5 answer.

Demographic data

1. Gender: Female/Male/Rather not say answer.
2. Age: numeric answer
3. Profession in the health field: specialized doctor/Resident doctor/General doctor/Community doctor/Nurse/Paramedic/Cardiopulmonary technician/Licensed in bioanalysis/Student/Other answer.
4. Area where you work: Internal Medicine/Infectious Diseases/Pulmonology/Surgery/Obstetrics and gynecology/Pediatrics/Public health/Anatomopathology/Laboratory/ICU/Emergency/Imagenology/Other answer.
5. Establishment where you work: short answer.
6. State where you are located: drop down options with all Venezuelan states (24).
7. Do you work on the first line against COVID-19? (having direct contact with suspicious or confirmed cases at the triage, consults, taking and/or analyzing samples, transport, treatment, care or hospitalization): Yes/No answer.
8. Where did you acquire knowledge about COVID-19? (Check as many as needed):

Training at the health center where I work.

Scientific literature.

Colleagues.

TV/radio/newspapers.

Instagram/Twitter/WhatsApp/Facebook.

Friends/Neighbors.

Other:

1. The center where you work has fit areas for the management of COVID-19 patients (distancing in a single room or at least separation of 1 meter between beds, correct management for biohazard waste, among others): 1 (never) to 5 (always) answer.
